# Cost-effectiveness of decompression according to Gill versus instrumented spondylodesis in the treatment of sciatica due to low grade spondylolytic spondylolisthesis: A prospective randomised controlled trial [NTR1300]

**DOI:** 10.1186/1471-2474-9-128

**Published:** 2008-09-28

**Authors:** Mark P Arts, Marco JT Verstegen, Ronald Brand, Bart W Koes, M Elske van den Akker, Wilco C Peul

**Affiliations:** 1 Department of Neurosurgery, Leiden University Medical Center, Leiden, The Netherlands; 2 Department of Neurosurgery, Medical Center Haaglanden, The Hague, The Netherlands; 3 Department of Medical Statistics & Bioinformatics, Leiden University Medical Center, Leiden, The Netherlands; 4 Department of General Practice, Erasmus Medical Center, Rotterdam, The Netherlands; 5 Department of Medical Decision Analysis, Leiden University Medical Center, Leiden, The Netherlands; 6 Spine Intervention Prognostic Study (SIPS) Group Leiden – The Hague

## Abstract

**Background:**

Nerve root decompression with instrumented spondylodesis is the most frequently performed surgical procedure in the treatment of patients with symptomatic low-grade spondylolytic spondylolisthesis. Nerve root decompression without instrumented fusion, i.e. Gill's procedure, is an alternative and less invasive approach. A comparative cost-effectiveness study has not been performed yet. We present the design of a randomised controlled trial on cost-effectiveness of decompression according to Gill versus instrumented spondylodesis.

**Methods/design:**

All patients (age between 18 and 70 years) with sciatica or neurogenic claudication lasting more than 3 months due to spondylolytic spondylolisthesis grade I or II, are eligible for inclusion. Patients will be randomly allocated to nerve root decompression according to Gill, either unilateral or bilateral, or pedicle screw fixation with interbody fusion. The main primary outcome measure is the functional assessment of the patient measured with the Roland Disability Questionnaire for Sciatica at 12 weeks and 2 years. Other primary outcome measures are perceived recovery and intensity of leg pain and low back pain. The secondary outcome measures include, incidence of re-operations, complications, serum creatine phosphokinase, quality of life, medical consumption, costs, absenteeism, work perception, depression and anxiety, and treatment preference. The study is a randomised prospective multicenter trial in which two surgical techniques are compared in a parallel group design. Patients and research nurse will not be blinded during the follow-up period of 2 years.

**Discussion:**

Currently, nerve root decompression with instrumented fusion is the golden standard in the surgical treatment of low-grade spondylolytic spondylolisthesis, although scientific proof justifying instrumented spondylodesis over simple decompression is lacking. This trial is designed to elucidate the controversy in best surgical treatment of symptomatic patients with low-grade spondylolytic spondylolisthesis.

## Background

Spondylolytic spondylolisthesis is an anterior slip of one vertebral body onto another caused by a discontinuity (lysis) of the pars interarticularis, also called the isthmus. As a result of the pseudarthrosis, fibrocartilaginous tissue develops at the isthmic site. Although most people with spondylolytic spondylolisthesis are asymptomatic, patients may present with low back pain, sciatica, neurogenic claudication, or a combination due to nerve root compression in the parapedicular course under the fibrocartilaginous mass of the pseudo-joint, or at the neuroforamen between the pedicle and slipped disc.

Treatment of patients with symptomatic spondylolytic spondylolisthesis consists of nonsurgical management or surgery whereby surgery yields better outcome [1]. Surgical management comprises primary reconstruction of the pars interarticularis defect, decompression of the nerve root without fusion, decompression with (instrumented) spondylodesis, or a combination. Transpedicular fixation with interbody fusion is the most frequently advocated surgical technique [2-9].

A less invasive approach, in which nerve root decompression is performed without fusion, has been described by Gill [10]. The operation according to Gill consists of removing the loose lamina and excising the fibrocartilaginous tissue in order to decompress the nerve root. Various studies have reported satisfactory results [11-13]. Recently, we have presented the long-term results of 42 patients treated with bilateral nerve root decompression according to Gill or unilateral decompression; i.e. hemi-Gill. At 11 years after surgery improvement of leg pain was reported in 88%, and 71% documented good result in terms of patient satisfaction [14].

Although most surgeons perform nerve root decompression with instrumented fusion in the treatment of spondylolytic spondylolisthesis, scientific proof justifying instrumented spondylodesis over simple decompression is lacking. The clinical results of instrumented and noninstrumented spondylodesis seem comparable with decompression according to Gill; 60 to 90% of the patients have good results [4,6,15-20]. The main arguments that favour intercorporal instrumented fusion are enlargement of the neuroforamen to relieve nerve root compression, and prevention of progressive slippage. Instrumented spondylodesis, on the other hand, constitutes major surgery with considerable blood loss, longer operation time, and significant complication rates which correlate with the extent of fusion [19,21,22].

Gill's procedure is a less invasive alternative technique to instrumented fusion. The operation time is shorter and the paraspinal muscle injury is less extensive. Patients may have less surgery-related low back pain and mobilise quickly, indicating short hospitalisation, fast recovery and early resumption to work. In addition, costs and complications related to instrumented surgery are avoided. However, secondary instrumented surgery might be necessary in patients with recurrent or persisting leg pain due to foraminal nerve root compression [14].

The controversy in the treatment of low grade spondylolytic spondylolisthesis justifies a randomised controlled trial comparing instrumented spondylodesis with nerve root decompression alone. The purpose of our study, the Sciatica-Gill trial, is to assess the clinical outcome and cost effectiveness of both surgical strategies on the short term (12 weeks) and long term (2 years). There will be a trade-off between persisting or recurrent leg pain in the Gill group and higher complication rates and costs in the spondylodesis group. Secondly, various subgroups of patients will be identified who may benefit primarily from one of the allocated surgical treatments.

## Methods/design

We designed a multicenter randomised controlled cost-effectiveness trial on the treatment of low-grade spondylolytic spondylolisthesis in which two surgical techniques are compared in a parallel group design. The primary outcome measure is the Roland Disability Questionnaire (RDQ) for sciatica. The follow-up period will last 2 years (with extension to 5 years). In order to collect enough patients, a multicenter design is necessary. The study protocol is approved in all participating hospitals by the Medical Ethics Committee.

We hypothesise that nerve root decompression according to Gill is more (cost-)effective on the short term (12 weeks) and equally (cost-)effective on the long term (2 years). Equal effectiveness is defined as maximal 4 points difference on the RDQ score between decompression and instrumented fusion. Moreover, we will identify subgroup of patients who may benefit more of one of the allocated treatments.

### Patients

All patients between 18 and 70 years with sciatica or neurogenic claudication lasting more than 12 weeks, based on imaging proven (MRI and CT-scan) spondylolytic spondylolisthesis grade I or II, are eligible for this study. During the first visit to the neurological outpatient clinic, the patient's history will be taken and a standardized neurological examination will be performed. During this visit the surgeon will inform the patient on the pathology of spondylolytic spondylolisthesis and controversies in best surgical treatment for this condition. The study will be explained to the patient and in case of a positive reaction an appointment will be made with one of the research nurses as soon as possible. After informed consent, the patient will be randomised during the first visit at the research nurse once the in- and exclusion criteria are met (table 1). Various questionnaires, outcome measures and baseline variables will be recorded.

**Table 1:** Selection criteria for trial eligibility

***Inclusion criteria:***
• Age 18–70 years
• Spondylolytic spondylolisthesis grade I or II
• Persistent sciatica or neurogenic claudication, with or without low back pain, lasting more than 3 months
• Operation indication
• Informed consent

***Exclusion criteria:***
• Spondylolytic spondylolisthesis grade III or IV
• Herniated disc on affected level, which requires discectomy
• Low back pain only
• Instability on dynamic X-ray (>3 mm)
• Progressive spondylolisthesis
• Previous surgery on affected level
• Extreme obesitas (BMI > 35)
• Severe osteoporosis/chronic use of steroids
• Severe comorbidity/contraindication for surgery
• Planned migration to another country on the short term
• Inadequate knowledge of Dutch language
• Pregnancy

### Randomisation procedure

Patients will be randomly allocated to nerve root decompression according to Gill, either unilateral or bilateral, or instrumented spondylodesis. The patient, surgeon, physiotherapist and research nurse are not blinded for the allocated treatment. Randomisation lists are prepared for every participating hospital separately using variable sized blocks of randomly allocated treatments to patients, sequentially numbered in order of inclusion, to ensure near-equal distribution of patients over the two randomisations arms in the hospitals. The data manager, who is not involved in the selection and allocation of patients, will prepare coded, sealed envelopes containing the treatment allocation. During the first visit of the research nurse, the envelope will be opened in presence of the patient and appointments will be made for the hospital admission. Surgery will be performed within 6 weeks after randomisation. Eligible patients who have a strong preference for one of the allocated surgical techniques, and are not willing to be randomised but are consenting to participation in the study, will be followed as a separate cohort.

Patients, who are randomised for nerve root decompression according to Gill and have persisting leg pain on the first follow-up moment at 12 weeks, are offered secondary instrumented spondylodesis. These patients will be analysed in the Gill group conform the intent-to-treat principle.

### Intervention

Patients will be randomised in nerve root decompression according to Gill, unilateral or bilateral (Group A), and decompression combined with instrumented spondylodesis (Group B).

#### (A) Decompression without spondylodesis; (hemi)Gill's procedure

A lumbosacral midline incision will be made and the paravertebral muscles will be dissected unilateral or bilateral, depending on the patients' symptoms. The spondylolytic lamina and the inferior articular process are removed, together with fibrocartilaginous mass of the pseudo-joint. In case of a unilateral procedure a vertical hemi-laminectomy will be performed; i.e. hemi-Gill. The affected nerve root(s) will be decompressed adequately, implicating a reduction of the superior articular process when necessary. The wound will be closed in layers with a suction drain when necessary.

#### (B) Decompression with instrumented spondylodesis

The above-described bilateral decompression will be performed in all patients. After performing bilateral discectomy, pedicle screws will be placed in the affected segment under fluoroscopic control. The intercorporal space will be filled with either a cage or autologous iliac crest, depending on the surgeon's preference. Sagittal alignment will be achieved and the screws will be fixed to the rods under slight compression. The wound will be closed in layers with a suction drain when necessary.

Various surgical findings will be documented: severity of nerve compression (none, moderate, severe, extreme), localisation of nerve root compression (lateral recess, parapedicular under the pseudo-joint, neuroforamen between slipped disc and pedicle, or a combination), aspect of the nerve root (normal, purple), aspect of the disc (normal, bulging, protrusion, extrusion, sequester, slipped disc), dural tear, nerve root damage, blood loss, duration of the procedure, judgment on placement of pedicle screws and intercorporal grafts. Postoperative time of mobilisation and length of hospital stay will also be documented.

### Baseline data

Baseline assessment includes demographics, hobbies, sports, work status, smoking status, back pain history, medical history and co-morbidity, body mass index, and neurological signs and symptoms. The patient's satisfaction at work will be registered. The treatment preference of patient, surgeon and research nurse will be assessed on a 5-point scale ranging from "strong preference for Gill's procedure" to "strong preference for instrumented spondylodesis". In any case all (clinical) outcome assessments will also be measured at baseline as far as they are applicable at that moment.

### Outcome assessment

Most symptomatic patients with spondylolytic spondylolisthesis report leg pain, low back pain and disability to perform normal daily activities. We will assess the below described validated outcome parameters. Patients will not be informed about their previous scores. Follow-up examination will take place at 6, 12, 26, 52, 104 and 260 weeks after randomisation. Additionally, at 3, 156 and 208 weeks after randomisation, questionnaires will be sent by mail (table 2).

**Table 2:** Data collection and outcome measures.

***Time in weeks***	*X*	*0*	***3***	*6*	*12*	*26*	*52*	*104*	***156***	***208***	*260*
Preferred treatment of patient/surgeon/RN	v										
Neurological examination	v			v	v	v	V	v			v
Randomisation	v										
Operation		v									
Perceived recovery according to patient (Likert)	v		**v**	v	v	v	V	v	**v**	**v**	v
Perceived recovery according to surgeon/RN (Likert)				v	v	v	V	v			v
Macnab	v			v	v	v	V	v			v
HADS	v			v	v	v	V	v			v
Severity of complaints (VAS)	v	v	**v**	v	v	v	V	v	**v**	**v**	v
Roland Disability Questionnaire	v	v	**v**	v	v	v	V	v	**v**	**v**	v
Karasek	v			v	v	v	V	v	**v**	**v**	v
SF-36	v			v	v	v	V	v	**v**	**v**	v
EuroQol	v	v	**v**	v	v	v	V	v	**v**	**v**	v
Patient diary	v		**v**	v	v	v	V	v	**v**	**v**	v
CPK		v									
X-ray	v				v		V	v			v
MRI	v						V				
CT	v				v		V				
(Re)-operation			**v**	v	v	v	V	v	**v**	**v**	v
Complications		v			v		V	v			v

X is randomisation and 0 is operation. On week 3, 156, and 208 (bold columns), questionnaires will be sent by mail

### Primary outcome measure

Roland Disability Questionnaire for Sciatica (RDQ). This illness-specific 23-item functional assessment questionnaire is frequently used for low back pain and sciatica [23]. Scores range from 0 to 23, reflecting a simple unweighted sum of items endorsed by the respondent. High score reflects severe disabling pain. Recovery of pain is defined as improvement of 11 points or more from baseline [24]. This self-report measure of physical disability due to low back and leg pain has shown high level of internal consistency, construct validity, and responsiveness [24,25]. The RDQ is the main primary outcome measure in this trial.

### Secondary outcome measures

#### 1) Perceived recovery

An important secondary outcome measure is perceived recovery according to the 7-point Likert scale, ranging from "complete recovery" to "worse than ever". This outcome scale has been used in previous studies and appears to be valid and responsive to change [26,27]. "Complete recovery" and "almost complete recovery" are considered good results. Perceived recovery will be documented by the patient, surgeon and research nurse.

#### 2) Visual Analogue Scale (VAS) of leg pain and low back pain

This parameter will measure the experienced pain intensity in the leg and low back during the week before visiting the research nurse. Pain will be assessed on a horizontal 100 mm scale varying from 0 mm, "no pain", to 100 mm, "worst pain imaginable". Patients do not see the results of earlier assessments and will score the pain experienced at the visit. Reliability, validity and responsiveness of VAS have been shown [28].

#### 3) Macnab classification of surgical result

Patients will be evaluated by the surgeon and research nurse using the Macnab classification [29] ranging from "excellent no pain; no restriction of activity, "good; occasional back or leg pain of sufficient severity to interfere with the patient's ability to do his or her normal work, or to enjoy leisure activity", "fair; improved functional capacity, but handicapped by intermittent pain of sufficient severity to curtail or modify work or leisure activities", and "poor; no improvement or insufficient improvement to enable increase in activities; further operative intervention required".

#### 4) The Short-Form 36 (SF 36)

The SF 36 is a generic health status questionnaire, which can be easily filled out at home. It consist of 36 questions measuring health on 8 multi-item dimensions with a score ranging from 0 (unhealthy) to100 points (optimal health) covering physical functioning, physical restrictions, emotional restrictions, social functioning, somatic pain, general mental health, vitality, and general health perception. This questionnaire has been used frequently and is validated in studies on low back pain and surgery [30,31].

#### 5) The Karasek Job Content Questionnaire

The work perception of the patient will be assessed according a 5-point standardized scale according to the Karasek Job Content Questionnaire. This questionnaire is designed to measure psychological demands, decision latitude, social support, physical demands, and job insecurity [32].

#### 6) Hospital Anxiety Depression Scale (HADS)

This questionnaire consists of 7 questions concerning depression and anxiety. The score ranges from 0 to 21. A high score indicates more complaints: a score between 0 and 7 excludes depression and anxiety, a score between 8 and 10 suggests probable depression/anxiety, and a score between 11 and 21 indicates depression/anxiety [33].

#### 7) Utility measurements

The EuroQol (EQ-5D) measures 5 dimensions (mobility, self-care, daily activities, pain/discomfort, anxiety/depression), on a 3 point scale (no, some, or extreme problems). For each health state described by the patients, a utility score can be calculated that reflects society's valuation of that health state. The Dutch tariff for the EQ-5D will be used [34]. Similarly, SF-6D utilities will be calculated from the SF-36 profiles [35]. Whereas the EQ-5D and SF-6D provide society's assessment of the patients' health, the patients themselves will also assess their own health on VAS, ranging from 0.0 (as bad as death) to 1.0 (optimal health). Both the EQ-5D and the VAS will be reported in questionnaires filled out at home.

#### 8) Radiological examinations

Preoperatively, all patient will undergo dynamic radiographs, CT and MRI. The exact slip percentage and the slip angle will be measured on plain X-ray. The localisation of nerve root compression (lateral recess, parapedicular underneath pseudojoint, neuroforamen between pedicle or slipped disc, or combination) and the amount of nerve root compression will be quantified on MRI. We have developed the following grading scale based on the epidural fat reduction in the neuroforamen: 0–25% fat reduction (grade 1), 25–50% fat reduction (grade 2), 50–75% fat reduction (grade 3), and more than 75% fat reduction (grade 4). In addition, the diameter of the neuroforamen will be measured on sagittal CT reconstructions. During the follow-up, dynamic X-rays, CT and MRI will be repeated (table 2).

### Other outcome measures

#### 1) Costs

The direct medical costs of hospital admission (fixed costs per admission, and variable costs per admission day) and surgery (including personnel and implants) will be estimated in all participating hospitals for cost-analysis. Using cost diaries, the patients will register other medical care (including physiotherapy, visits to general practitioners and medical specialists, nursing care and medication) and non-medical costs (including out-of-pocket expenses, domestic help and absenteeism). Each diary will cover 3 months and the research nurse will go through the diary with the patient on every follow-up visit, throughout the study period of 2 years. Costs will be calculated using standard prices, including time and travel costs [36]. Absenteeism will be valued according to the friction cost method.

#### 2) Incidence of re-operations

In previous studies concerning spine surgery the incidence of re-operations has been used to assess outcome. Also in this study, we will assess the incidence of re-operation. Every surgical re-exploration in both groups will be considered as re-operation.

#### 3) Complications

Side effects or complications that are ascribed to the treatment are recorded by the patients, their treating physicians and the research nurses.

#### 4) Serum creatine phosphokinase (CPK)

Creatine phosphokinase is a known marker of muscle damage. Previous study has demonstrated a dose-response relation between the extend of surgery and CPK values [37]. Therefore, we will determine the serum CPK before surgery and 1 day after surgery in both groups. Whether CPK is correlated with postoperative low back pain and clinical outcome will be determined as well.

### Sample size

The sample size is calculated on the basis of the RDQ both at both short term and long term follow-up. A difference in effectiveness is considered clinically relevant whenever patients with nerve root decompression alone will obtain at least 20% difference in RDQ score. Instrumented spondylodesis is assumed to result in at least 60% good outcome at 12 weeks. This means that 80% of the patients treated with nerve root decompression alone should have good results in order to proof superiority of Gill's procedure. Treatment is considered to be justified in case of improvement of at least 7 points on the RDQ score; 20% difference corresponds to 8.4 points improvement on in the RDQ score. With a power of 90% and a two-tailed significance level of 0.05, 220 patients with symptomatic spondylolytic spondylolisthesis grade I and II are needed (110 patients in both treatment groups, including 10% loss to follow-up). The numbers used for this sample size calculation are retrieved from the 1 and 5-years results of the Maine Lumbar Spine Study [38,39].

### Statistical analysis

Baseline comparability will be analysed by descriptive statistics to determine whether randomisation was successful. Whenever necessary to remove residual confounding or simply in order to increase power, adjustments for baseline variables will be performed in the analyses. Differences in outcome measures between both groups, together with 95% confidence intervals, will be calculated. We stipulate in advance that we will also analyze a model containing the interaction of follow-up time and randomisation group; if the latter is significant at the 5% level, the conclusion of "unequality of treatments" follows; if the latter is non-significant, the treatment effect will be calculated on the basis of a model without group*time interaction. All data will be analysed according to the "intention-to-treat principle". We state a priori that we will perform as a subgroup analysis, the evaluation of the interaction between the treatment allocation and both the grading of the disease (grade I versus grade II spondylolisthesis) as well as the localisation of nerve root compression (underneath the pseudo-joint or between pedicle and slipped disc). We will conclude that the treatment effect depends on the value of those variables if the interaction is significant at the 5% level. If the interaction is non-significant, we will quantify the main effect of these variables in the usual way. In addition, explorative subgroup analyses will be performed, based on the interaction between the randomization variable and various different baseline variables (table 3). Data will be stored via the internet-based secure data management system "ProMISe" of the department of Medical Statistics and Bioinformatics. The analyses will be carried out using appropriate statistical software (e.g. SPSS).

**Table 3:** Selected prognostic variables for subgroup analysis

***Demographic variables:***
• Women versus men
• Age < 40 years versus > 40 years
• Smoking versus non-smoking
***Anamnestic and neurological variables:***
• Influence of back extension versus no influence
• Predominant low back pain versus leg pain
• Quetelet index < 25 versus > 25
***Radiological variables:***
• Low disc height versus high disc height
• Nerve root compression underneath pseudojoint versus nerve root compression between pedicle and slipped disc
• Grade I versus grade II spondylolytic spondylolisthesis
• Small cross section versus large cross section of neuroforamen
• Low grade versus high grade epidural fat

## Discussion

In this article, the rational and design of a RCT on cost-effectiveness of two surgical strategies for sciatica caused by low-grade spondylolytic spondylolisthesis are described. To our knowledge this is the first RCT comparing nerve root decompression according to Gill versus instrumented spondylodesis. Based on the literature, the clinical results of nerve root decompression alone and instrumented spondylodesis seem equivalent, although solid conclusions cannot be made because of various methodological flaws.

The Sciatica-Gill trial is designed to elucidate the controversy in best surgical treatment of patients with symptomatic spondylolytic spondylolisthesis grade I and II. Gill's procedure is a less invasive procedure, patients can return to work quickly and instrumented related complications are avoided. However, secondary instrumented spondylodesis might be necessary in patients with persistent or recurrent leg pain. Currently, most surgeons advocate primary instrumented fusion. Arguments in favour of intercorporal spondylodesis are prevention of progressive slippage, enlargement of the neuroforamen, reduction of dynamic nerve root compression due to instability, and possible influence of low back pain. Whether more invasive surgery with higher costs and complications warrants instrumented spondylodesis as primary treatment, can be debated. Therefore we postulate that nerve root decompression according to Gill is more (cost-) effective on the short term and equally (cost-) effective on the long term, compared to instrumented spondylodesis. Subgroup analysis might identify patient groups who will benefit more from one of the treatments. The inclusion period will run until medio 2010 and follow-up measurements will be completed in 2012.

## Abbreviations

RCT: Randomised Controlled Trial; VAS: Visual Analogue Scale; CRF: Case Record Form; RDQ: Roland Disability Questionnaire.

## Competing interests

The authors declare that they have no competing interests.

## Authors' contributions

MA designed the protocol and is the supervisor of MV. MV is the coordinator and primary investigator of the trial. RB is responsible for the sample size calculation, has contributed to the case record forms and is responsible for the implementation of the trial data management using the ProMISe software; he is also the biostatistician for this trial, with the exception of the cost-effectiveness analysis. BK is the epidemiological supervisor. EA is responsible for the design of the cost-effectiveness analysis. WP is the principal investigator and responsible budget holder. All authors participated in the trial design and coordination. All authors read and approved the final manuscript.

## Pre-publication history

The pre-publication history for this paper can be accessed here:


